# Numerical Optimization and Map-Based Manipulation With a Quadrupole Electromagnetic Actuated System

**DOI:** 10.3389/fnbot.2022.859996

**Published:** 2022-03-17

**Authors:** Weicheng Ma, Zhijie Huan, Min Xu

**Affiliations:** School of Electrical Engineering and Automation, Xiamen University of Technology, Xiamen, China

**Keywords:** electromagnetic actuated system, system optimization, micro-manipulation, map-based control, numerical simulation

## Abstract

Electromagnetic actuation is a new technique for non-invasive manipulation, which provides wireless and controllable power source for magnetic micro-/nano-particles. This technique shows great potential in the field of precise mechanics, environment protection, and biomedical engineering. In this paper, a new quadrupole electromagnetic actuated system was constructed, which was composed of four electromagnetic coils, each coil being actuated by an independent DC power supplier. The magnetic field distribution in the workspace was obtained through finite element modeling and numerical simulation *via* COMSOL software, as well as the effect of the current flow through the coil in the field distribution. Moreover, parameters of the electromagnetic system were optimized through parametric modeling analysis. A magnetic field map was constructed for rapidly solving the desired driving current from the required magnetic flux density. Experiments were conducted to manipulate a micro-particle along the desired circular path. The proposed work provides theoretical references and numerical fundamentals for the control of magnetic particle in future.

## Introduction

Micro-manipulation aims to control the movement and assembly of micro-particles in the target workspace for some specific applications including micro-processing (Li et al., 2015), environmental governance (Wang et al., 2015), and drug targeted delivery (Gao et al., 2016). Since the size of the manipulated particle is in micro-scale, it is hard to directly integrate a traditional embedded energy supply device. Thus, non-invasive mechanisms are introduced for micro-manipulation technology, including dielectrophoresis (Chu et al., 2015; Huan et al., 2016) generated by non-uniform electric field, optical tweezers (Cheah et al., 2014; Xie et al., 2019) induced by focused laser beam, and magnetic driving force (Ma et al., 2017; Niu et al., 2017; Meng et al., 2019) generated by gradient magnetic field. Compared with dielectrophoresis and optical tweezers, magnetic actuated technology has its advantages in biological compatibility and micro-flexibility (Pankhurst et al., 2003; Ma et al., 2020), which has been widely investigated in recent years.

The actuators of magnetic actuated systems are usually composed of permanent magnets or electromagnetic coils. Permanent magnets have relatively high magnetic energy product per unit volume, which could efficiently and economically generate magnetic field of large strength (Mahoney and Abbott, 2014). However, the magnetic field induced by permanent magnet is difficult to be removed beyond the working state. The variation of the magnetic field could only be achieved by adjusting magnetic pole interval in the workspace (Wright et al., 2017). The magnetic field generated by the electromagnetic coil could be controlled with variable currents flowing in the coils (Yesin et al., 2006). The electromagnetic system could provide a remote controllable driving force for magnetic particles in a relatively large workspace, which is convenient for system modeling and control law design (Wang et al., 2018). Electromagnetic actuated system has been reported by many researchers. Kummer et al. introduced an electromagnetic driven system consisting of eight electromagnetic coils with iron cores, which could provide three dimensions of translation and two dimensions of rotation for magnetic particles in the workspace (Kummer et al., 2010). An electromagnetic driven system composed of two pairs of saddle coils with different geometric parameters was designed by Jeon et al. (2010), which could enlarge the effective workspace for the manipulation. Li et al. proposed an electromagnetic actuated system with four intersecting electromagnetic coils in the plane. The end of the core near the workspace was designed as a probe, and the other end of the core was attached with a thin iron sheet (Li et al., 2020). This could not only increase the generated magnetic field gradient but also enlarge the effective workspace.

In this paper, a new quadrupole electromagnetic actuated system is proposed, which could provide real-time adjustable magnetic field distribution in its workspace with external programmable current suppliers. Structure was designed to achieve an adjustable workspace. Parameters of the system were optimized through parametric modeling and finite element simulation. Moreover, based on the magnetic field data value of the discrete reference points, a magnetic field map in the workspace was constructed to obtain the inverse solution of the required current value rapidly in real time. Experiments were conducted for manipulation of micro-particles with the proposed setup. The proposed work provides a foundation for manipulating micro-particles and improving the control response speed of the electromagnetic actuated system.

## Materials and Methods

### System Design

**Figure 1:**
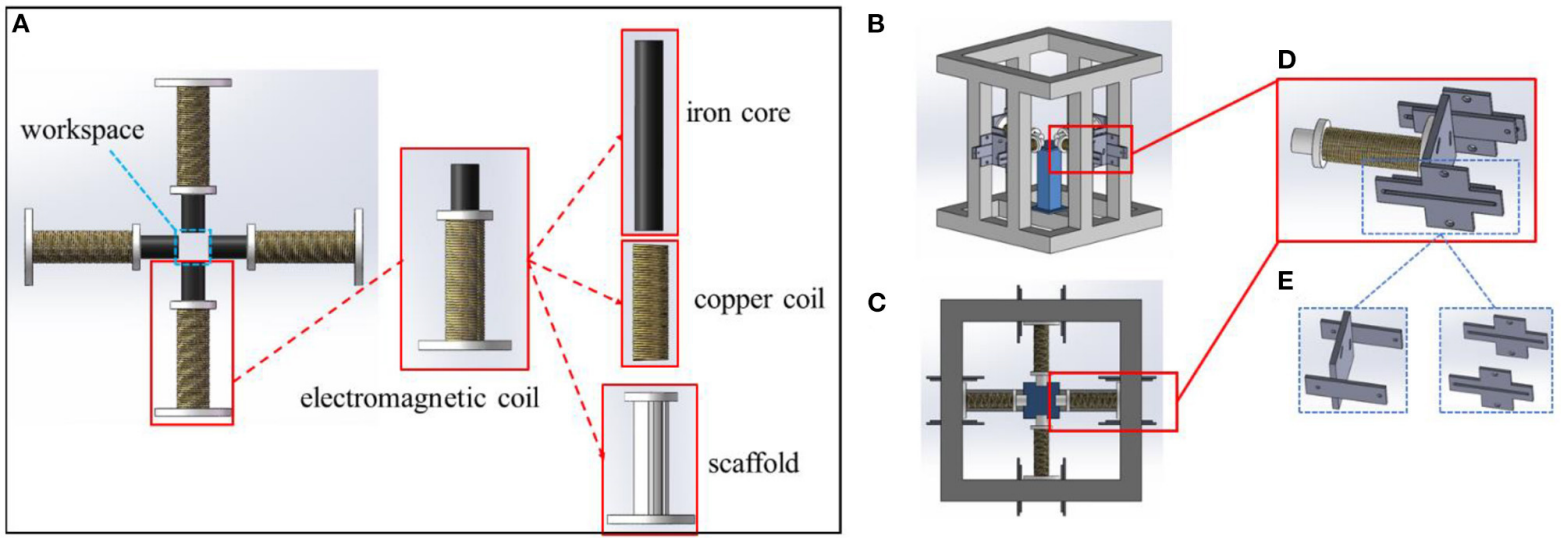
Configuration of the electromagnetic actuated system. **(A)** Combination of the electromagnetic coils; **(B)** structure of the entire system; **(C)** top view of the system; **(D)** fixation for the electromagnetic coils; **(E)** H-type bracket and guide rail.

A quadrupole electromagnetic actuated system was designed to generate a gradient magnetic field for manipulating micro-particles. As shown in Figure 1A, the basic structure of the system consists of four electromagnetic coils, which is constituted by scaffold, copper coil, and iron core. The coils are further connected to the power suppliers. Thus, a square magnetic workspace is provided in the center of the system.

As shown in Figures 1B,C, the entire system is settled within an aluminum frame. Each electromagnetic coil is fixed with an adjustable supporter, as shown in Figure 1D. As illustrated in Figure 1E, the connection supporters consist of one H-type bracket and two guide rails. With these supporters, the relative distance of the four electromagnetic coils is alterable. Since the generated magnetic field is related to the structure of the system, the induced magnetic field could be further adjusted according to certain applications.

In order to enhance the induced magnetic field, DT4-core was utilized for the electromagnetic coils, which has excellent electromagnetic performance. Meanwhile, the characteristic of electrical pure iron DT4 is also friendly with manufacturing and could be fabricated according to the designed structure easily. The DT4-core has a large area of liner part which could be used for our electromagnetic system. DT4 has relatively low coercive force (H_c_ ≤ 96 A/m) and high magnetic conductivity (μ ≥ 7.5 × 10^−3^ H/m), which benefits the generation of a precise magnetic field.

To avoid electromagnetic interference, the entire setup was settled on a non-magnetic optical vibration isolation platform. A commercial three-dimensional (3D) printer (M3D) was used to fabricate the core-scaffold with standard polylactic acid (PLA) filaments. The 3D models of scaffold were first built with Solidworks. Each scaffold was designed with several grooves along the axial direction for promoting heat dissipation of coils during the operating process. Enameled copper wires were wrapped and stacked around the scaffold forming electromagnetic coils, with a diameter of 1.3 mm. The electromagnetic coils were fixed on the aluminum profile bracket constituting a quadrupole electromagnetic system. An aluminum stage was installed right under the workspace, which could be used for placing the experimental chip. The observation system was composed of optical microscope, charge-coupled devices (CCD) camera, and computer, in order to record the movement of micro-particles. Programmable power suppliers (GWinstek GPD 3303S) were connected to each coil to control the current input of the manipulation system.

### Electromagnetic Actuation Mechanism

As a magnetic micro-particle suspended in the gradient magnetic field, both force and torque will be induced on the particle which are related to the magnetic property of micro-particles and the distribution of the magnetic field. The induced force and torque could be obtained from the following equations:


(1) $$\begin{array}{l} \vec{F}=\left(\vec{Q}•\nabla \right)\vec{B} \end{array}$$

(2) $$\begin{array}{l} \vec{T}=\vec{Q}\times \vec{B} \end{array}$$

where, $\vec{B}={\left(B_{x},B_{y},B_{z}\right)}^{T}$ is the magnetic flux density of the magnetic field in Cartesian coordinate system, $\vec{Q}={\left(Q_{x},Q_{y},Q_{z}\right)}^{T}$ is the magnetic moment of the magnetic particle, and ∇ is the gradient operator.

For a liner, isotropic, and homogeneous magnetic particle with volume *V*_*p*_ and susceptibility χ, the magnetic moment $\vec{Q}$ could be defined as (Huan et al., 2021b):


(3) $$\begin{array}{l} \vec{Q}=V_{p}\frac{\chi }{\mu_{0}\left(1+\chi \right)}\vec{B} \end{array}$$

where, ${\;\mu }_{0}\;=\;4\pi \times 10^{-7}\;Tm/A$ is the free-space permeability.

Substituting (3) into (1), the magnetic force could be written as:


(4) $$\begin{array}{l} \vec{F}=V_{p}\frac{\chi }{\mu_{0}\left(1+\chi \right)}\left(\vec{B}•\nabla \right)\vec{B} \end{array}$$

Since the magnetic field of the electromagnetic coils is generated by current-carrying coils, the magnetic flux density could be calculated with the Biot-Savart law. With current $\vec{I}$, the induced magnetic flux density could be given as:


(5) $$\begin{array}{l} \vec{B}=\frac{\mu_{0}I}{4\pi }\int \frac{d\vec{l}\times (\vec{P}-{\vec{P}}^{\prime })}{{\left|\vec{P}-{\vec{P}}^{\prime }\right|}^{3}}\int =\vec{\zeta_{B}}I \end{array}$$

where, $\vec{P}={\left[p_{x},p_{y},p_{z}\right]}^{T}$ is the position of the manipulated micro-particle, ${\vec{P}}^{\prime }$ is the magnetic field source position, $d\vec{l}$ is the current element in the magnetic coils, and $\vec{\zeta_{B}}=\frac{\mu_{0}}{4\pi }\int \frac{d\vec{l}\times (\vec{P}-{\vec{P}}^{\prime })}{{\left|\vec{P}-{\vec{P}}^{\prime }\right|}^{3}}$ is related to the position of the particle.

According to Equations (4) and (5), the magnetic force induced on the micro-particle placed in the magnetic field is related to the coil current $\vec{I}$ and the particle position $\vec{P}$. Generated magnetic field for a certain magnetic coil is linearly related to the coil current, which could be obtained from Equation (5). As we have four electromagnetic coils, the magnetic field in position $\vec{P}$ is a vector superposition of each coil's contribution, which could be written as:


(6) $$\begin{array}{l} \vec{B_{p}}\left(x,y,z\right)=\overset{4}{\underset{n=1}{\sum }}\vec{B_{np}}\left(x,y,z\right)=\overset{4}{\underset{n=1}{\sum }}\left(\vec{\zeta_{nB}}I_{n}\right) \end{array}$$

where, $\vec{B_{np}}\left(x,y,z\right)$ is the contribution in position $\vec{P}$ of any one electromagnetic coil, and *I*_*n*_ is the current input for each coil, separately.

## Results and Discussion

### Parameter Optimization

In order to evaluate and optimize the magnetic field distribution of the proposed electromagnetic system, an FEM model was constructed *via* the COMSOL software. The 3D model structure of the quadrupole magnetic system was first built in Solidworks. Four coils were aligned orthogonal to each other in X-Y plan. The model was then converted and imported into COMSOL for further analysis.

**Figure 2:**
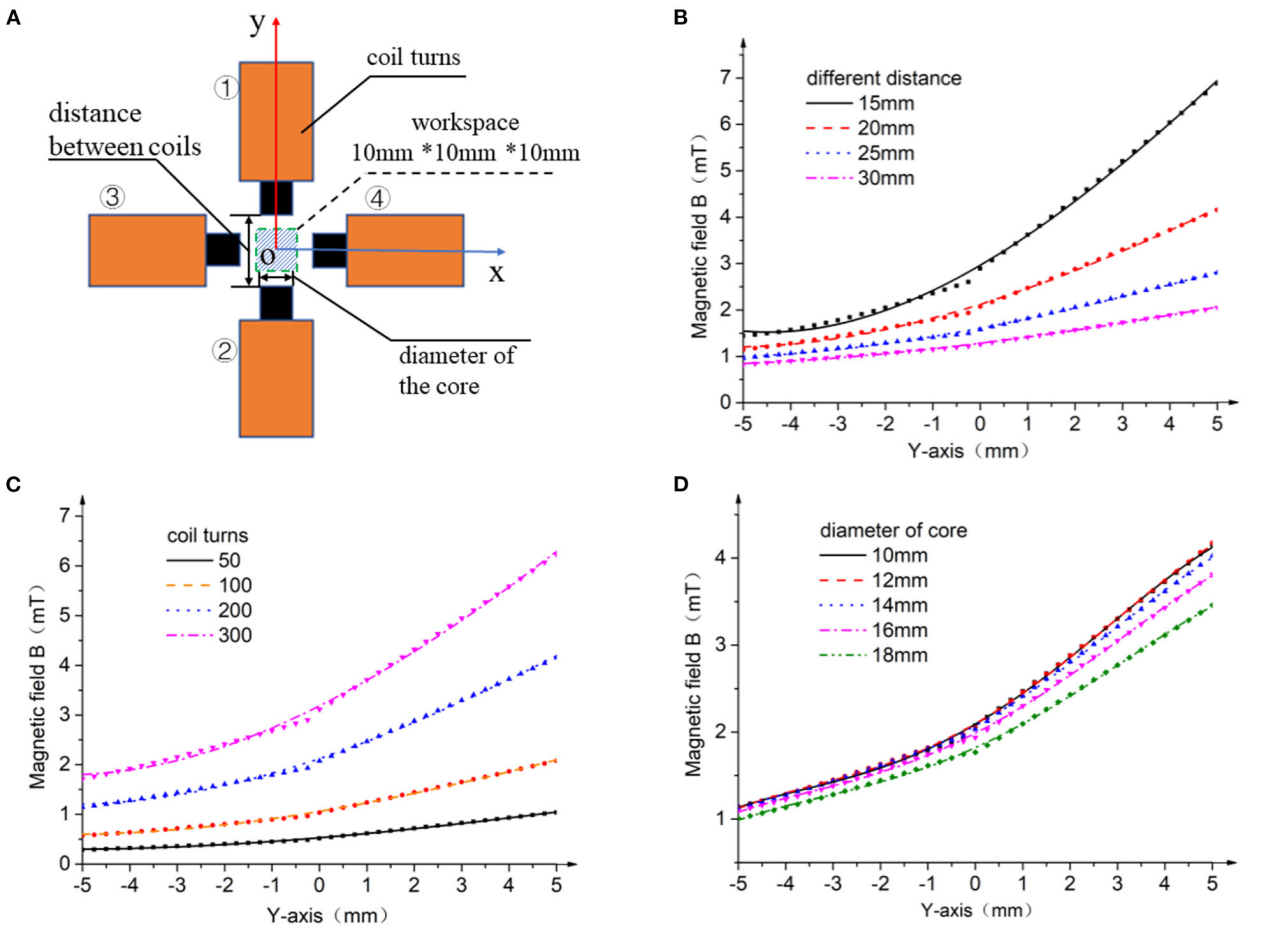
Parameter optimization of the electromagnetic system. **(A)** Parameterized model; **(B)** magnetic field distribution with different distance between coils; **(C)** magnetic field distribution with different coil turns; **(D)** magnetic field distribution with different diameters of iron core.

As given in Equation (6), the magnetic field within the workspace could be calculated through the principle of vector superposition. Thus, the magnetic field generated by a single coil was analyzed in the first place. According to the Biot-Savart law, the induced magnetic field is related to the design parameters of the electromagnetic coil system, such as diameter of core, distance between coils, and number of coil turns. Parameterized model of the system was built for optimization, as shown in Figure 2A. In the simulation, coil 1 is applied with 1A current, and there is no current going through the other coils. Magnetic field distribution in the central axis of coil 1 is exported. Simulation results with different parameters are illustrated in Figures 2A–C.

As illustrated in Figure 2B, different distances between two concentric coils were set for the simulation. The distribution curves of magnetic field show that the closer to the core, the larger is the gradient of magnetic field. Moreover, as the distance between two concentric coils increases, the magnetic field around the center of workspace and its gradient obviously decrease. For better driving characteristics, the distance between two concentric coils should be small enough. However, when we reduce the distance, the workspace would also be compressed. Thus, in order to guarantee a 10 mm × 10 mm valid workspace, 20 mm was chosen as the distance between two concentric coils.

The number of coil turns is also an important factor for magnetic field. The magnetic field distribution along Y-axis was analyzed with different coil turns, as shown in Figure 2C. The magnetic field and its gradient could be enhanced by increasing the number of coil turns. This result could also be predicted theoretically from Equation (6). Nevertheless, for the limitation of size, coils are spooled multi-layered which will cause heat dissipation. To avoid excess thermal effect during long running operations, the number of coil turns was chosen as 200.

Figure 2D summarizes the magnetic field distribution with different diameters of iron core. As we reduce the diameters, the magnetic field and its gradient in the central axis gradually increase. A smaller iron core is preferred according to the simulation results. In addition, if the diameter of iron core is smaller than 10 mm, the difficulty of manufacturing and coil spooling process is increased significantly. Based on overall consideration, the diameter of the iron core was set as 10 mm.

### Magnetic Field Analysis

The parameters of the system were optimized along the central axis. In order to investigate the magnetic field distribution in the workspace, the model was constructed according to Figure 2A. A current value of 1 A was then applied to each electromagnetic coil. It should be noted that the current flows in the opposite direction in coil 1 and coil 3, as well as in coil 2 and coil 4, while the current flow is in the same direction for coil 1 and coil 4.

**Figure 3:**
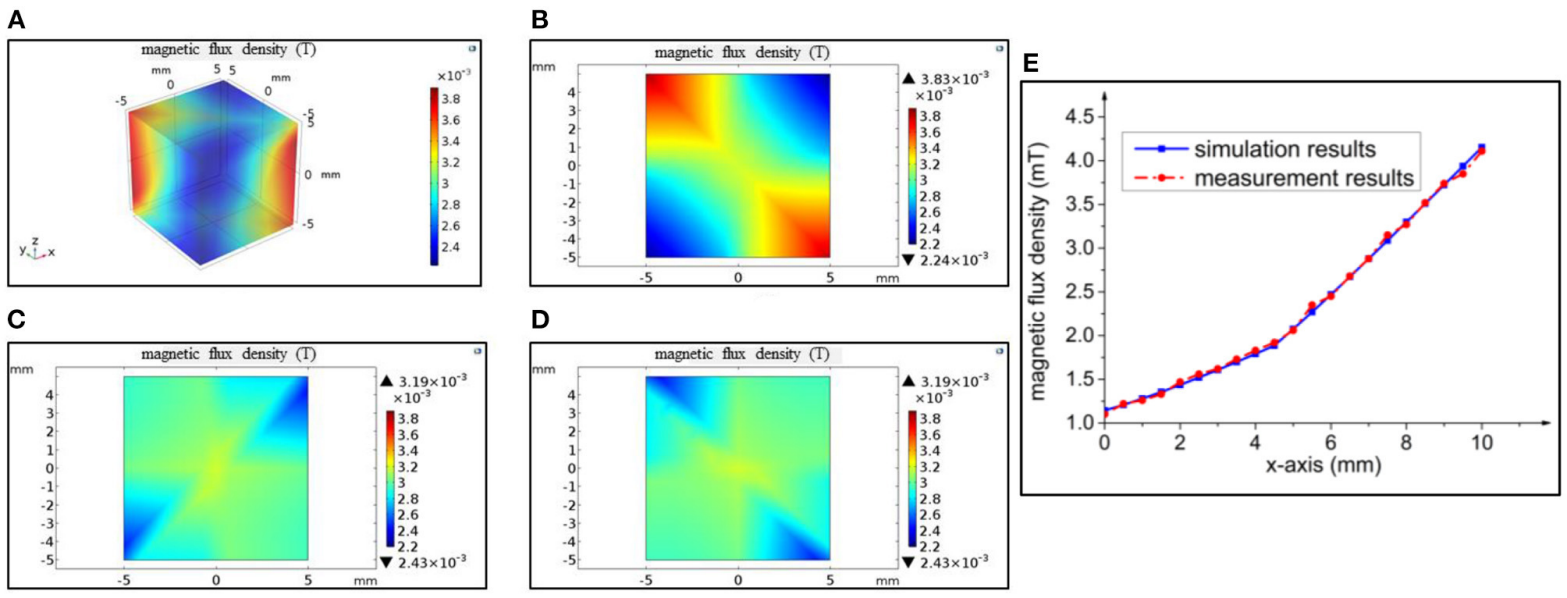
Simulation of the electromagnetic system in the workspace. **(A)** Three-dimensional (3D) magnetic field distribution; **(B)** magnetic field distribution in XY-plane; **(C)** magnetic field distribution in XZ-plane; **(D)** magnetic field distribution in YZ-plane; **(E)** comparison between magnetic fields evaluated with COMSOL and measured with gaussmeter in X-axis.

The magnetic field distribution in the 10 mm × 10 mm × 10 mm workspace could be calculated with finite element analysis. The 3D steady state magnetic field distribution is proposed in Figure 3A. In order to show more details, the magnetic field distributions in two-dimensional (2D) cross section are exported in Figures 3B–D. It is evident that the magnetic field in each 2D plane is symmetrically distributed. As illustrated in Figure 3B, the magnetic field strength near the coils with opposite currents is much higher compared with the rest of the region. As we can observe from Figures 3C,D, the field strength decreases along the Z-axis significantly. Thus, the magnetic field in the center plane is the strongest, which is the most suitable for target manipulation. Our experiments could be further conduced within the center plane of the workspace.

After the simulation, we reconstructed the system with optimized parameters. A gaussmeter (LZ-610) was used to measure the magnetic field distribution along the central axis of coil 1, to which a current of 1 A was applied. The probe of the gaussmeter was fixed on a triaxial micromotion platform, and the value was recorded with a distance interval of 0.5 mm. Figure 3E shows the comparison between the results from software simulation and real measurement for coil 1. The comparison result shows that the simulation results are consistent with the experimental results. Some minor error could be introduced by the system disturbance and manufacturing process.

### Map Construction

According to Equations (4) and (5), during the manipulation of micro-particle, the applied current could be calculated from the required driving force, which is given by the control algorithm. However, partial differential equations should be solved in this process and the solution is non-unique. The complex solution process could introduce delay for the manipulation. One possible solution is to prepare a magnetic field database for the workspace, with which we could find out the specific current directly from the given magnetic field strength. As illustrated in Equation (5), the magnetic flux density is in direct proportion to the applied current in case the magnetic core is not saturated. Furthermore, the overall magnetic field depends on the vector superposition of each electromagnetic coil's contribution. We just need to construct a unit-current magnetic field map for each coil.

In order to construct the unit-current magnetic field map, the values of the magnetic flux density for each node in FEM model were exported from COMSOL. The discrete reference points were treated as the data source. During the manipulation, the target particle could be anywhere in the workspace, whose position was captured from the visual system. To determine magnetic flux density in the target position, spherical search method and inverse distance weighting algorithm were utilized. For an arbitrary point in the workspace, a spherical region with variable diameter was used for searching the data sample around the target. The diameter is an auto-increment value ensuring enough neighboring sample data are found.

The magnetic flux density in target position could be given as:


(7) $$\begin{array}{l} \vec{B_{0}}=\underset{a=x,y,z}{\sum }\vec{B_{0a}} \end{array}$$

where, *B*_0*a*_ is target magnetic flux density in axis_X, axis_Y, and axis_Z, respectively. The value of each axis could be fitted by the distance inverse weight method. The interpolation weights were set as reciprocal of distance between the target point and the sample data points.


(8) $$\begin{array}{l} B_{0a}=\frac{\sum_{i=1}^{n}\frac{1}{d_{ia}}B_{ia}}{\sum_{i=1}^{n}\frac{1}{d_{ia}}} \end{array}$$

where, *B*_*ia*_ is the magnetic flux density in certain axis of sample data point.

### Manipulation Experiment

**Figure 4:**
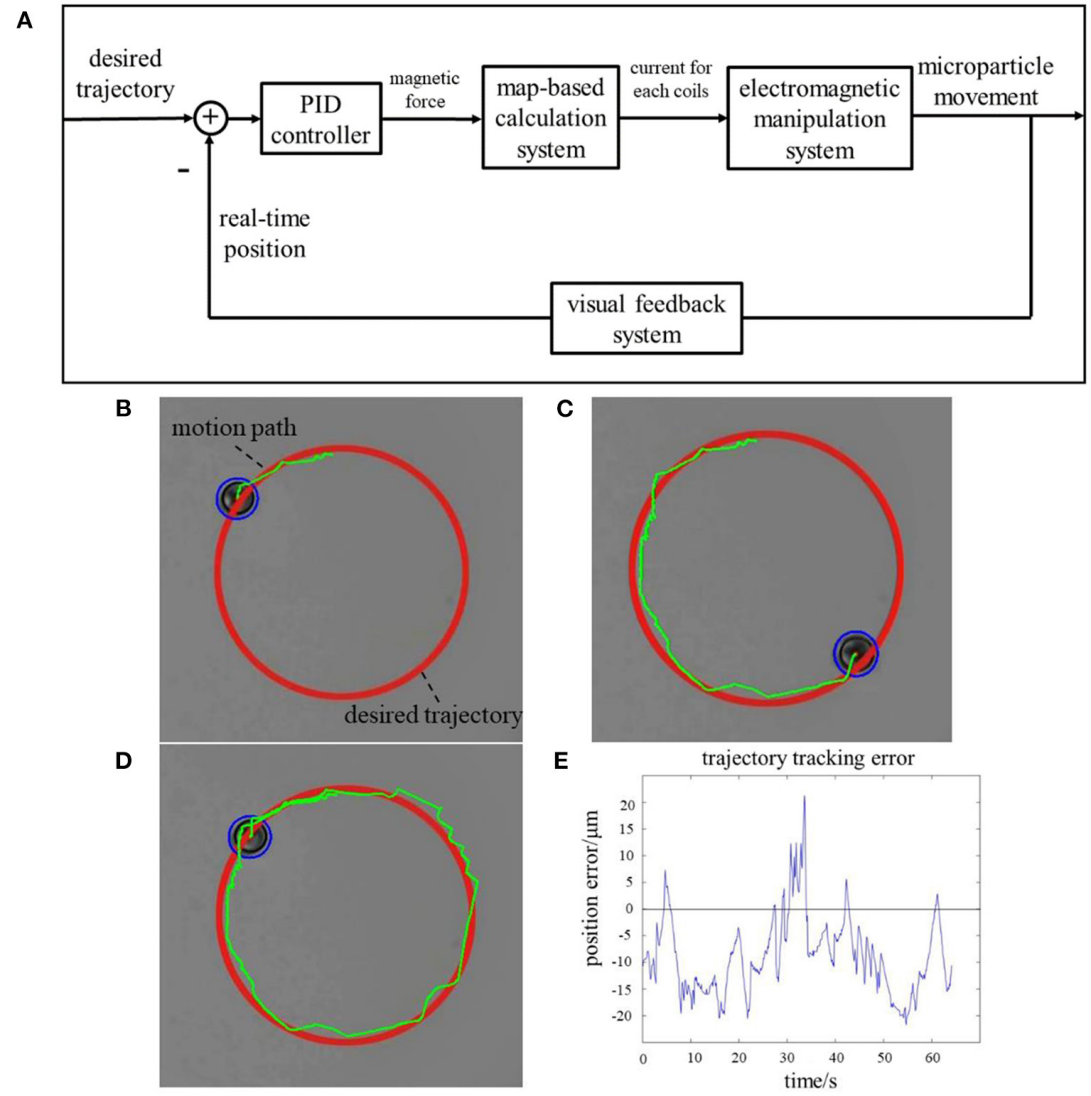
**(A)** Micro-particle control schematic. Captured images for manipulating a micro-particle along desired circular path at different moments: **(B)** at *t* = 5 s; **(C)** at *t* = 25 s; **(D)** at *t* = 40 s; **(E)** trajectory tracking error.

Experiments were performed with the proposed system to manipulate magnetic micro-particle. A micro-particle along a desired circular path was tracked with a PID controller. The control schematic diagram is shown in Figure 4A. The real-time position of the micro-particle was located by image process with real-time captured images. Desired magnetic force was obtained with PID controller. Since the input for electromagnetic manipulation system is the current for each coil, the desired current should be calculated with map-based calculation system from the desired magnetic flux density. Magnetic micro-particle was then actuated accordingly.

The radius of the micro-particle was 5 μm, with a density of 1.1 g/cm^3^. During the manipulation, the micro-particle was suspended in salt water, which has a similar density, to avoid sinking or rising. The desired trajectory was provided with a software interface. Real-time position of the target micro-particle was detected by image process. Currents for each electromagnetic coils were obtained by the position error-based feedback controller. Required magnetic force could be generated within the workspace. The micro-particle could then be manipulated to track the provided trajectory automatically. Figures 4B–D illustrates the captured images for manipulation process, in 5 s, 25 s, and 40 s, separately. Trajectory tracking error was performed as in Figure 4E. The maximum error did not exceed 20 μm, which indicates that the whole system demonstrated good control effect.

## Conclusion

In this paper, a new quadrupole electromagnetic actuated system has been presented to generate a gradient magnetic field for manipulating micro-particles. The overall structure of the system was constructed. The magnetic field distribution was simulated with COMSOL. Furthermore, parameters of the electromagnetic coils were optimized for enhancing the magnetic flux density within the workspace. The magnetic field map of the workspace was constructed *via* spherical search method and inverse distance weighting algorithm. Experiments were conducted with the map-based manipulation system. The proposed work provides theoretical references and numerical fundamental for the control of magnetic particle. Future work could focus on the precise control method (Zhong and Xu, 2021; Zhong et al., 2021) for micro-particle manipulation within this electromagnetic actuated system.

## Data Availability Statement

The original contributions presented in the study are included in the article/supplementary material, further inquiries can be directed to the corresponding author/s.

## Author Contributions

WM and ZH: conceptualization, methodology, validation, data analysis, and writing—original draft. WM and MX: materials development and writing—review and editing. All authors have read and agreed to the published version of the manuscript.

## Funding

This work was supported by National Natural Science Foundation of China (NSFC) under grant numbers 61903315 and 62003285 and the Natural Science Foundation of Fujian Province under grant numbers 2019J05124, 2019J01869, and 2020J01285.

## Conflict of Interest

The authors declare that the research was conducted in the absence of any commercial or financial relationships that could be construed as a potential conflict of interest.

## Publisher's Note

All claims expressed in this article are solely those of the authors and do not necessarily represent those of their affiliated organizations, or those of the publisher, the editors and the reviewers. Any product that may be evaluated in this article, or claim that may be made by its manufacturer, is not guaranteed or endorsed by the publisher.
